# Barriers to help-seeking in Israeli Arab minority adolescents with mental health problems: results from the Galilee study

**DOI:** 10.1186/s13584-019-0315-7

**Published:** 2019-05-23

**Authors:** Raida Daeem, Ivonne Mansbach-Kleinfeld, Ilana Farbstein, Alan Apter, Rasha Elias, Anneke Ifrah, Gabriel Chodick, Silvana Fennig

**Affiliations:** 1 Sackler Faculty of Medicine, Tel Aviv University, Tel Aviv, Israel; 2 Child and Adolescent Mental Health Department, Ziv Medical Center, 13100 Zefat, Israel; 3 The Feinberg Child Study Center, Schneider Medical Center for Children in Israel, 49202 Petach Tikvah, Israel; 4 Department of Psychiatry, Schneider Children’s Medical Center of Israel, 49202 Petach Tikva, Israel; 5 Ruppin Academic Center, Netanya, Israel; 6 Inter-Disciplinary Center, Herzliya, Israel; 7 Israel Center for Disease Control, Gertner Institute, Sheba Medical Center, 5265601 Tel Hashomer, Israel; 8 School of Public Health, Tel Aviv University, Tel Aviv, Israel; 9 Epidemiology and Data Base, MaccabiTech, Tel Aviv, Israel

**Keywords:** Adolescents, Mental health, Barriers to help-seeking, Stigma, Accessibility, Muslim, Druze, Israel, SDQ, DAWBA

## Abstract

**Background:**

The Galilee Study assessed mental health service needs among Israeli Muslim and Druze adolescents and their mothers. Studies show that mothers of adolescents belonging to the Arab minority have much lower help-seeking rates than Jewish mothers. This paper examines mothers’ structural and cultural barriers to help-seeking.

**Methods:**

All 9th grade students living in 5 towns representative of Muslim and Druze localities in northern Israel, were eligible for the study and 1639 (69.3%) obtained parental agreement and participated. Emotional or behavioral problem were assessed in the classroom using the Strengths and Difficulties Questionnaire. A total of 704 adolescent-mother dyads participated in the follow-up, and were interviewed at home, using the Development and Well Being Assessment inventory, the Composite Barriers to Help-Seeking Questionnaire, the General Health Questionnaire − 12, the Subjective Feelings of Discrimination Index and socio-demographic questions. Pearson χ^2^ test and multivariate binary logistic regressions were performed to analyze mothers’ consultation rates by risk factors. Exploratory factor analysis was performed to identify underlying factors and assess construct validity of the Composite Barriers to Help-Seeking Questionnaire, and also mean scores and standard deviations for the distinct scales were calculated.

**Results:**

More mothers of adolescents with a mental disorder than those without a mental disorder consulted a professional or school source (39.7% vs. 20.5%; χ^2^ = 45.636; *p* = < 0.001). The most important barriers to help-seeking were those related to “Accessibility”, followed by barriers related to the belief that “Treatment is detrimental” and to the possibility of “Reprisal by authorities”. Barriers related to “Stigma” and “Distrust of professionals” had the lowest means scores**.** Differences by ethnicity/religion were found.

**Conclusions:**

Structural barriers related to lack of access, were considered the main obstacle to help-seeking in this Israeli Arab minority population. Cultural barriers such as stigma were considered of secondary importance. Structural barriers could be reduced by increasing the number of accessible public mental health clinics in the minority localities, a responsibility of the Ministry of Health and the HMOs. Information campaigns and psychoeducation for parents would help reduce other barriers to mental health treatment.

## Introduction

The prevalence of mental disorders among adolescents, according to a worldwide pooled estimate, is 13.4% [1]. The Israeli Survey of Mental Health among Adolescents (ISMEHA), which included a nation-wide representative sample of 14–17 year old Israeli adolescents, reported a prevalence rate of 8.1% for internalizing disorders and of 4.8% for externalizing disorders [2]. The Galilee Study, focusing on Muslim and Druze Arab minority adolescents in Israel reported prevalence rates of internalizing and externalizing disorders of 15.8 and 4.2%, respectively, among Muslim adolescents and rates of internalizing and externalizing disorders of 5.9 and 5.5%, respectively, among Druze adolescents [3]. Many studies show that only half of those children and adolescents with a mental disorder receive any care and that less than one-fourth are cared for by a mental health specialist [4–11]. The percentage of untreated adolescents is known to be larger among minorities [5, 9, 12–19], and among socio-economically disadvantaged adolescents [20–22].

The ISMEHA showed that parents belonging to the Arab minority in Israel were much less likely to seek help when concerned for their children’s mental health than parents from the majority population [9], although rates of mental disorders of Jewish and Arab adolescents did not differ significantly [2]. A possible reason for the disparity in help-seeking could be lack of public mental health services. A mapping of public mental health clinics for children and adolescents in Israel showed that in the Northern and the Southern District, where over 80% of the Israeli Arab population lives [23], only 16.8 and 4.3% of Israeli Arab children in need of care, respectively, were cared for by the mental health clinics [24].

Several factors may explain what prevents different population groups from accessing mental health care for themselves and their children. According to Cauce and colleagues, help-seeking, whether from formal or informal sources, is influenced by structural, organizational, cultural and social contexts [25]. Structural contexts shape conditions of access—the number, type, affordability and quality of psychosocial supports available in a community and those resources necessary to access those further away [26–30]. Different organizational settings serve different populations: community-based mental health centers are usually accessible to parents, whereas teachers and school staff are an important and available source for consultation by both parents and the children themselves [5, 9, 25, 31–35]. Cultural factors influence perceptions of need for services, which rely upon one’s explanatory framework for psychosocial problems—beliefs about their origin, how they can best be resolved, and what constitutes a problem that is serious enough to necessitate assistance from a professional helper [14]. In addition, negative consequences and perceptions, including stigma [36, 37], or negative effects of treatment or mental health services [38], have been associated with reduced help seeking behaviors. The Child and Adolescent Service Assessment (CASA) [39], in its assessment of obstacles to help-seeking, includes additional factors such as fear, dislike, or distrust of professionals; prior negative experiences with professionals; shame or anticipation of negative reactions; anticipation that the child may be removed from the home or anticipation of loss of parental rights; and lack of accessibility (information about services, bureaucratic hindrances, language compatibility). Reardon and colleagues [11] in their systematic review of 144 quantitative and qualitative studies concerning barriers to care, found that the main barriers were systemic-structural barriers such as accessibility, cost of services, waiting periods and getting a referral. Following in order of importance were barriers related to views and attitudes towards services, in particular feeling not listened to or dismissed/ blamed by professionals. These were followed by barriers related to loss of trust in professionals and fears concerning the negative consequences of treatment, fear associated with treatment itself, the detrimental impact of perceived negative attitudes of others on help seeking, as well as personal discomfort about mental health [11].

A number of concerns led us to further examine these obstacles. First, the low percentage of Israeli Arab mothers of children with mental disorders who consulted a professional source – 9% according to the ISMEHA [9]. Second, the need to inform the health authorities responsible for supervising and managing the Israeli Mental Health Reform approved as law in 2015, regarding ways to improve service provision to minority Arab Palestinian citizens of Israel (Arabs in Israel) in need of mental health care through the HMOs. The Arabs in Israel are an indigenous population, constituting about 21% of all Israeli citizens and 25.5% of those below 19 years of age. Over 80% of Arabs in Israel are Muslims, and the rest are mainly Druze and Christian [23], and they are over-represented in all the indicators of poverty, distress and underdevelopment, with high unemployment rates and school drop-out rates [40]. The Druze comprise a traditional and conservative Arabic-speaking cultural group [41] who participate in the Israeli military service. Druze males’ main employment is with the security forces. This improves their economic status and increases their adoption of norms of the Jewish majority [42]. The Muslim Israeli citizens, on the other hand, are a non-assimilated minority, mainly due to the continuing state of conflict between Israel and the Arab world, which has placed them in the status of a hostile minority outside the national consensus [43].

Considering the urgent need for mental health care for adolescents, the aim of the present study was to identify the barriers to mental health care among Muslim and Druze minority adolescents and their mothers. We posed several questions that needed clarification. They were: Which barriers to help-seeking are considered to be most important by mothers? Do these perceptions differ by ethnicity or type of disorder suffered by the adolescent? In what ways are mothers’ perceived barriers to help-seeking different according to whether they consulted or not? The goal of this study is to better understand obstacles to help-seeking for mental health concerns, in particular structural obstacles related to accessibility to services and cultural factors associated with stigma.

## Methods

### The study population

Five localities representative of the Muslim and Druze towns and cities in northern Israel insofar as population size, geographic location and ethno-national composition, were chosen to participate in the study. The 2012–2013 cohort of 9th grade students in these five Arab localities (*N* = 2366) was included in the study, excluding those who had dropped out, were non-school attenders, or were studying in out-of-town schools.

### Instruments and measurements

- *Strengths and Difficulties Questionnaire (SDQ)* – Self-reported Arabic version was used to assess emotional and behavioral problems of the adolescents (http://www.sdqinfo.com). The SDQ, a screening instrument designed for evaluating functioning in 4–17 year old children and adolescents [44], includes 25 items covering four clinical domains: hyperactivity-inattention, emotional symptoms, peer-relationship and conduct problems, and one pro-social behavior domain. The psychometric properties of the SDQ in Arabic have shown to be satisfactory [45, 46].
- *Development and Well-Being Assessment (DAWBA)* – Arabic version – (http://www.dawba.info), [45, 47], was used to diagnose mental disorders. DAWBA is a multi-informant package of questionnaires, interviews and rating techniques that generate ICD-10 and DSM-IV psychiatric diagnoses for children aged 5–17. These include internalizing disorders (separation anxiety, specific phobias, social phobia, panic attacks and agoraphobia, post-traumatic stress, compulsion and obsessions, generalized anxiety and depression and deliberate self-harm), externalizing disorders (hyperactivity-inattention and awkward and troublesome behavior), eating disorders, autistic spectrum and other disorders. In our study questions relating to troublesome behaviors (e.g., whether the child has lied, stolen or been questioned by the police), were censored by the Israeli Ministry of Education and excluded from the questionnaire, purportedly to prevent students’ self-incrimination.
- *The General Health Questionnaire-12 item version (GHQ-12)*, assesses mother’s risk of developing a psychiatric disorder as defined by distress and inability to carry out normal functions [48]. Mothers in the highest 33% of the distribution were considered as being at high risk and those in the lowest 67% as being at low risk. The Arabic version has an internal reliability (Cronbach alpha) of .86 [49].
- *Subjective feelings of discrimination*: Four items adapted from the Public Regard Subscale of the Multidimensional Inventory of Black Identity [50], relating to how others respect one’s community, behave towards it, appreciate it and feel it contributes to the State of Israel were used. These four items were pooled into a “Feeling of Discrimination” Index (FDI) and categorized as high or low. The FDI had an internal reliability (Cronbach alpha) of 0.885.
- *Obstacles to professional help-seeking*: The Composite Barriers to Help-seeking Questionnaire (CoBaQ), includes 14 questions pertaining to 4 parameters taken from the Child and Adolescent Services Assessment (CASA) [39], and three additional questions pertaining to one parameter added by the Galilee Study’s research team. In all, the CoBaQ included five parameters and 17 items. The four *parameters* expressed in 14 statements taken from the “Perceptions of Barriers to Service” from CASA are the following: 1. Fear, dislike, or distrust of professionals or previous negative experience; 2. Self-consciousness or anticipation of negative reaction; 3. Anticipation of out of home placement or loss of parental rights; 4. Accessibility: Incomplete information, time spent, concerns about cost, problems with transportation, problems with bureaucratic delay, problems with availability, language. The fifth *parameter* added by the Galilee Study team was: Perception that treatment is not beneficial or helpful and might even damage.
- *Personal attitude towards help-seeking*: Mothers were asked to assess the statement: “If my child had emotional or behavioral problems I would seek help or treatment for him/her”. They could choose one of four options: 1) definitely agree; 2) partly agree; 3) partly disagree; 4) definitely disagree. Given that more than 87% of mothers declared they definitely agreed, options 2, 3 and 4 were categorized as “do not agree” and the analyses were carried out on a dichotomized item.
- The *socio-demographic data* reported by the mothers included: religion, family size, parental educational, marital status, whether the family was in the care of a welfare agency and whether the adolescent had a learning disability (LD).
- The *socio-economic level of the locality* in which the family lives was defined according to the ranking of local authorities published by the Central Bureau of Statistics of Israel [51]. The parameters used for this classification were: demography (median age, dependency rates and percentage of families with 4 or more children), education (average years of schooling of adults aged 25–54 and percentage with an academic degree), employment and benefits (percentage of salaried workers aged 15+, percentage of women aged 25–54 with no work-related income, percentage of employed earning double the average wage, percentage of workers earning less than minimum wage and percentage receiving benefits), standard of living (e.g. average monthly income and number of vehicles owned). The 255 Israeli local authorities are ranked and assigned to socio-economic clusters ranging from 1 (lowest) to 10 (highest). The localities included in this study belong to clusters 4, 3 and 2, as do nearly 90% of the Arab localities in Israel, and were classified into three socio-economic levels: a) medium (cluster 4), b) low (cluster 3), and c) very-low (cluster 2).
- *Help-seeking and consultation*: Mothers were asked whether they had ever spoken about the emotional, behavioral or social problems of their child with a professional or with any other source such as primary health practitioner, pediatrician, internal medicine or any other medical specialty, psychiatrist, psychologist, another mental health specialist (social worker, psychiatric nurse, art therapist, language therapist), alternative medicine specialist, school advisor, teacher, school nurse, school psychologist, special education teacher, any other school source, self-help group, internet support group, chat-room, religious leader, social worker from the welfare services, family members, friends and other non-professional adults. We included in the “consulted” category mothers who sought help from a health or mental health professional or a school source. Those who sought help from a family member, self-help groups, friends, or through the internet were not included in this category.

### The study design

The study included a screening stage in the classroom and a follow-up stage at home. Adolescents with higher probability of having an emotional or behavioral problem according to the SDQ were over-sampled, in order to increase statistical power and the robustness of the analyses. In each locality all students in the highest 25% of the SDQ score distribution were considered at high risk for an emotional or behavioral problem and included in the sample for the follow-up stage, together with a simple systematic sample of students in the lower 75% of the distribution.

### Procedures

Only students whose parents signed an informed consent and had turned it in were recruited and requested to complete the SDQ in the classroom between September 2012 and May 2013. The follow-up took place between October 2013 and May 2014, when adolescents and their mothers were interviewed face-to face at their home, simultaneously and independently, by two lay interviewers. The study was approved by the Ethics Committee of the Rabin Medical Center (Request No, 6339).

### Statistical analyses

Mothers’ consultation practices were analyzed by socio-demographic and health-related risk factors and psychosocial traits of adolescents, using Pearson χ2 test, with a significance level set at ≤0.05. Multivariable binary logistic regressions were performed with consulting a professional or school source as dependent variables. Logistic regression coefficients were transformed into odds ratios (OR) with 95% confidence intervals (CI). Internal consistency of the CoBaQ and distinct scales was assessed with Cronbach’s alpha. An exploratory factor analysis (EFA) was performed to identify underlying factors and assess construct validity of the CoBaQ [52]. Mean scores and standard deviations for the distinct CoBaQ scales were calculated.

All data were weighted to account for the sampling design in each locality and SQD category in order to generalize the study sample to the reference population as follows: The inverse sampling probability of each individual in the sample was divided by the mean of the inverse sampling probabilities of all individuals in the group to yield a weighting variable scaled such that the mean weight of all individuals is 1 and the weighted sample size equals the actual, unweighted sample size [53]. Statistical analyses were conducted using an IBM SPSS-21 module (IBM Corp. Released 2012. IBM SPSS Statistics for Windows, Version 21.0. Armonk, NY: IBM Corp.).

### Data collection and response rates

Data were collected in two stages. During the first stage, the screening stage, the total response rate was 69.3%. In the follow-up stage, each one of the four localities was assigned a different sampling fraction according to size, in order to include approximately the same number of subjects in each locality. Response rates were around 90%, except for Locality 4, where lack of street names and house numbers made finding the families difficult. Total response rate among located subjects was 92.3, and 84.5% when including refusals and not located in the “non-response” category.

Selected characteristics of adolescents who agreed to participate in the study (*N* = 1639), and of those who refused (*N* = 727), were compared. Among those who refused, there was a higher proportion of boys than among those who participated (59.4% vs. 42.5% respectively), and a higher proportion of students considered by teachers to be “low achievers” (34.7% vs. 21.9%, respectively). No differences were found by religion (Data not shown).

## Results

### Actual help-seeking practices

Overall, 24.4% of Muslim mothers and 20.7% of Druze mothers consulted a professional or school source. Among Muslim mothers of children with a mental disorder 37.3% consulted, while among those with no disorder 21.3%% consulted (χ2 = 24.49; *p* = .001). Among Druze mothers of children with a mental disorder 47.9% consulted, while those with no disorder 17.3% consulted (χ2 = 24.36; *p* = .000).

Table 1 presents mothers’ consultation rates by socio-demographic, health-related and psychosocial traits of adolescents. Mothers of boys had higher consultation rates than mothers of girls, mothers in the low socio-economic index had higher consultation rates than those in the medium or very low socio-economic indexes, mothers in families that were in care of a welfare agency had higher consultation rates than those who were not. Mothers of adolescents with an internalizing or externalizing disorder or a LD had higher consultation rates than mothers of those without a disorder or a LD, mothers with a high GHQ score had higher consultation rates than those with a low GHQ score and mothers of adolescents who felt uncomfortable at home had higher consultation rates than mothers of adolescents who felt comfortable at home.

**Table 1:** Socio-demographic, health-related and psychosocial traits of the study population and mothers’ consultation rates for mental health concerns (weighted numbers and percentages)

Variables	Total (*N* = 1639)		Mothers’ consultation rates				
	%	n	%	n/N	χ2;	df;	*p*
Socio-demographic variables							
Gender							
Boys	47.6	780	27.6	214/774	12.470;	1;	< 0.001
Girls	52.4	859	20.2	172/852			
Religion							
Muslim	70.0	1147	24.4	277/1135	2.455;	1;	.117
Druze	26.8	440	20.7	91/440			
Christian	3.2	52	–				
Socio-economic index							
Medium	15.3	251	8.0	20/251	58.206;	1;	.000
Low	46.9	768	31.0	234/756			
Very low	37.8	620	21.3	132/619			
Number of children in family							
1–3	25.9	420	25.1	105/419	0.861;	2;	.650
4–5	51.9	842	23.8	200/840			
6 or more	22.2	360	22.2	80/360			
In care of welfare							
Yes	21.4	340	34.1	116/340	23.636;	1;	.000
No	78.6	1248	21.4	267/1248			
Health-related variables							
Any mental disorder							
Yes	16.6	272	39.7	108/272	45.636;	1;	.000
No	83.4	1367	20.5	278/1354			
Internalizing disorder							
Yes	12.8	209	34.9	73/209	16.023;	1;	.000
No	87.2	1429	22.2	314/1417			
Externalizing disorder							
Yes	4.2	69	59.4	41/69	50.674;	1;	.000
No	95.8	1570	22.2	345/1557			
Learning disability							
Yes	4.9	79	55.1	43/78	44.863;	1;	.000
No	95.1	1545	22.1	341/1544			
GHQ of mother							
High	34.3	502	31.9	160/502	35.115;	1;	.000
Low	65.8	965	18.2	175/963			
Psycho-social variables							
Feeling discriminated Index							
High	23.8	370	26.7	96/360	2.920;	1;	.087
Low	76.2	1186	22.3	264/1183			
Feeling uncomfortable at home							
Yes	15.0	239	29.7	71/239	6.819;	1;	.009
No	85.0	1350	22.0	294/1338			
Total			23.6	386/1639			

In a logistic regression model we found that mothers of adolescents with an internalizing disorder were twice as likely to consult than those without an internalizing disorder, mothers of adolescents with an externalizing disorder were 5.7 times more likely to consult, those in welfare care were 2.2 times more likely to consult and mothers with a high GHQ score were 1.5 times more likely to consult than those with a low GHQ score. Religion and whether the adolescent feels uncomfortable at home were not found to be significantly related to maternal consultation over and above the effect of the other variables. (Table 2).

**Table 2:** Factors associated with mothers’ consulting a professional or school source for mental health concerns: Logistic regression analyses (OR and 95% CI)

Characteristics of the adolescent	Mothers consulted a professional or school source	
	OR	(95% CI)
Internalizing disorder		
Yes	2.05	(1.44–2.91)
No	1.00 [Reference]	
Externalizing disorder		
Yes	5.678	(3.28–9.82)
No	1.00 [Reference]	
Religion		
Muslim	1.16	(0.85–1.58)
Druze	1.00 [Reference]	
In welfare care		
Yes	2.24	(1.65–3.03)
No	1.00 [Reference]	
GHQ-12		
High	1.49	(1.11–1.99)
Low	1.00 [Reference]	
Feels uncomfortable at home		
Yes	1.28	(0.90–1.81)
No	1.00 [Reference]	

More mothers of adolescents with an externalizing disorder than an internalizing disorders consulted a professional or school source. Among adolescents with an internalizing disorder 34.8% of their mothers consulted and among adolescents with an externalizing disorder 59.4% of their mothers consulted. This yields a treatment gap of 65.2% for internalizing disorders and 40.6% for externalizing disorders. (Data not shown)

### Personal attitudes towards help-seeking

In all, 87.1% of mothers said they would seek help if their child had a mental disorder. We found no significant differences in the positive attitude of mothers towards help seeking according to whether their child had or did not have a disorder (90.3% vs 86.5%, respectively; χ2 = 2.88; *p* = 0.09). Among mothers of children with a mental disorder who had stated that they would definitely consult, 43.4% actually consulted while of those who had stated they would not consult, 11.5% did consult (χ2 = 9.90; *p* = 0.002) (Data not shown).

### Barriers to service use as measured by the Composite Barriers to Help-seeking Questionnaire (CoBaQ) cale

The 17 items included in the CoBaQ, the instrument used to assess obstacles to use of service among mothers in our study, were analyzed and their psychometric traits are presented.

#### Construct validity

Exploratory factor analysis was performed on the CoBaQ, in order to assess the construct validity of the instrument in our population. Principal components analysis (PCA) was used as the algorithm to maximize the variance explained [54]. Varimax rotation was applied as it maximizes the squared loadings of a factor, gives similar results to many oblique factorial solutions and produces an orthogonal solution [54]. To guide interpretation of the results of EFA, values in excess of 0.46 were used. For CFA statistical significance, model fit and significant loadings were used.

**Fig. 1:**
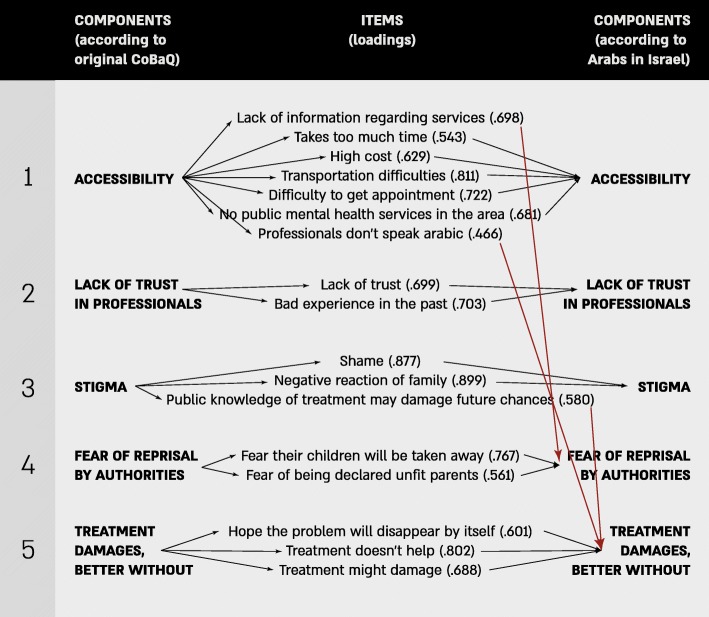
Exploratory factor analysis of the Composite Barriers to Help-seeking (CoBaQ) scales

Figure 1 shows the items originally included in each subscale and how these items were interpreted and re-categorized by our Arabic speaking population. Only the “Distrust in professionals” subscale loaded on the corresponding factors, while all other subscales were modified by our population. “Lack of information regarding services” and “Professionals don’t speak Arabic” – both items originally included as part of the “Accessibility” subscale loaded with the “Fear of reprisal” subscale and the “Treatment damages” subscale, respectively. “Public knowledge of treatment may damage future chances”, originally belonging to the “Stigma” subscale, loaded on the “Treatment damages” subscale.

#### Internal consistency

The CoBaQ (17 items), had a good internal consistency (α = .82) (See Table 3). The Stigma (anticipation of negative reactions) subscale had the highest reliability (α = .78), followed by the Accessibility subscale (α = .75), the Treatment is detrimental subscale (α = .62), the Fear of reprisal by authorities (loss of rights) subscale (α = .52) and lastly the Distrust in professionals subscale (α = .35).

**Table 3:** Internal consistency of the CoBaQ subscales: Original subscales and modified subscales according to loadings by Galilee Study’s population

Obstacles to Help-Seeking Sub-scales	Cronbach Alpha	
	Original CoBaQ Scale	CoBaQ according to Galilee population’s loadings
Stigma	.776	.895
Accessibility	.745	.713
Treatment damages	.621	.647
Fear of reprisal	.522	.614
Distrust professionals	.345	.345
Total Scale	.824	.824

However, when re-categorizing the subscales according to the factors that arose from the factorial analysis for this specific population (see below), we found slightly higher internal consistency for all subscales, except for the Accessibility subscale which became somewhat less consistent and the Distrust of professionals which remained the same. The Stigma subscale showed a high reliability (α = .90), followed by the Accessibility subscale (α = .71), followed by the Treatment damages subscale (α = .65), followed by the “Fear of reprisal” subscale (α = .61), and ending with the “Distrust of professionals” subscale (α = .35).

#### Mean scores

Mean scores for the CoBaQ subscales show that obstacles related to “Accessibility” had the highest mean score (M = 2.34; SD. = 2.6), followed by obstacles related to the belief that “Treatment is detrimental” (M = 1.32; SD = 2.2), followed by obstacles related to the possibility of “Reprisal by authorities” (M = 0.97; SD = 1.5), by obstacles related to “Stigma” (M = 0.55; SD = 1.2), and lastly by obstacles related to “Distrust of professionals” (M = 0.27; SD = 0.6).

### Mean CoBaQ scores by religion, type of mental disorder and help-seeking practices

**Fig. 2:**
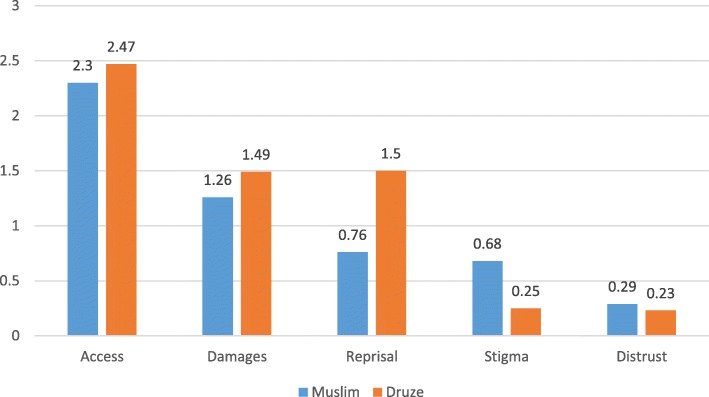
CoBaQ by religion

Mean scores for the CoBaQ subscales and comparisons by religion are presented in Fig. 2. No differences were found regarding obstacles related to “Accessibility” and to the belief that “Treatment damages”. However, we found that significantly more Druze than Muslim mothers saw the danger of “Reprisal by the authorities” as an obstacle to help-seeking (*F* = 85.15; *p* = .000) and significantly more Muslim than Druze saw “Stigma” and “Distrust in professionals” as obstacles to help-seeking (*F* = 41.44; *p* = .000, and *F* = 4.01; *p* = .046, respectively).

**Fig. 3:**
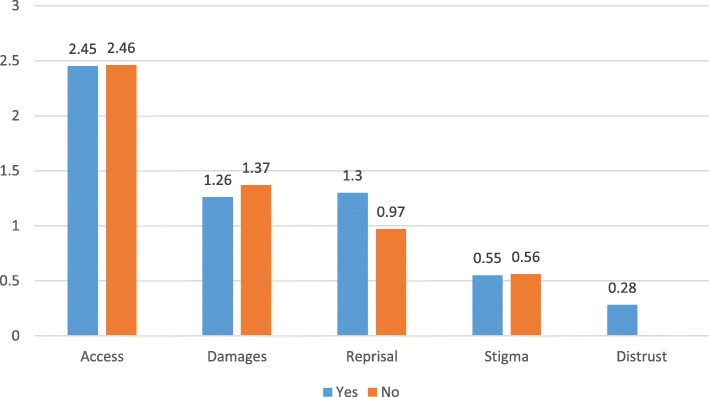
CoBaQ by whether the adolescent has an internalizing disorder

Figure 3 shows that more mothers of adolescents with an internalizing disorder thought that Reprisal or Distrust in professionals were obstacles to help-seeking (*F* = 22.02; *p* = .000 and *F* = 8.35; *p* = .004, respectively).

**Fig. 4:**
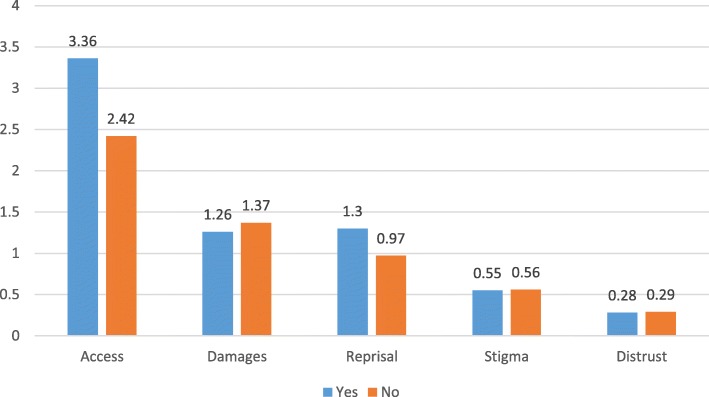
CoBaQ by whether the adolescent has an externalizing disorder

Figure 4 shows that more mothers of adolescents with an externalizing disorder than mothers of those without thought that obstacles related to accessibility were very important (*F* = 7.7; *p* = .006).

**Fig. 5:**
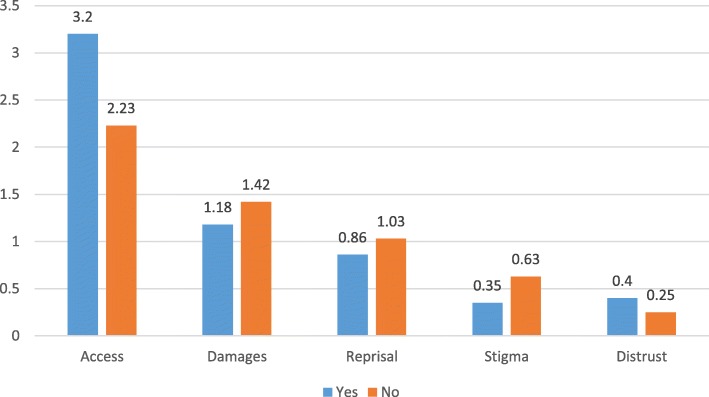
CoBaQ by whether or not the mother consulted

Figure 5 shows that mothers who did consult gave more importance to obstacles related to accessibility and to distrust of professionals than mothers who did not consult (*F* = 38.5; *p* = .000 and 17.0; *p* = .000, respectively). Mothers who did not consult gave more importance to obstacles related to stigma than mothers who did consult (*F* = 16.1; *p* = .000).

## Discussion

One of the main findings of the Galilee Study was that 37.3% of Muslim and 47.9% of Druze mothers of children with any mental disorder consulted a professional or school source, in comparison with 9% of Arab Israeli mothers in the ISMEHA study carried out in 2004–2005. The treatment gap was between 63% and 52% for any mental disorder, which is somewhat higher than the average in many parts of the world [5–8, 55], but is lower than the treatment gap found in the ISMEHA among Arab Israeli mothers [9]. There may be a number of possible explanations for this. One is the secular trend – the time elapsing between the studies, which may point to an increase in help-seeking among minority populations with increasing engagement with Israeli norms [56]. Another possible explanation is that the Galilee Study included a different population than that of the ISMEHA, as only Muslim and Druze living in the Northern and Haifa Districts were included, while the ISMEHA included also Bedouin populations in the Southern District, who have the lowest socio-economic and educational status of all Israeli citizens [57]. A third possible explanation for the increase in help-seeking among minority groups between 2004 and 2005 and 2012–2014 is that rates of mental disorders may be actually increasing among minority adolescents in Israel: while the ISMEHA found a prevalence of internalizing and externalizing disorders of 9.1 and 1.9%, respectively for Arabs in Israel [2] the Galilee study found a prevalence of internalizing and externalizing disorders of 15.8 and 4.2%, respectively, among Muslim adolescents who comprise nearly 80% of the Arabs in Israel [3].

A multivariable analysis indicated, as expected, that over and above the effect of other variables, mothers of children with an internalizing or externalizing disorder were 2 times and 5.7 times more likely, respectively, to seek help. Similar findings have been reported by others [5, 10, 33, 58]. Mothers in families in welfare care were 2.2 times more likely to seek help and mothers with a high GHQ score themselves were 1.5 times more likely to seek help. These findings may be related to the fact that both groups are familiar with or have had previous exposure to positive experiences with similar services. Our findings support studies that have found that the main variable promoting help-seeking is the existence of a mental disorder or mental distress in the adolescent [10, 31, 59–61], as well as those who have found that mental distress of the mother [60, 62, 63] and other problems in the household which require the intervention of the welfare agencies make help-seeking more likely. There is very likely an interplay between child and maternal health, their reciprocal relationships and cumulative disadvantage [64], which makes sense of the higher rates of mental problems among adolescents who experience poor parental emotional well-being and family adversity [65].

In our assessment of attitudes towards help-seeking we found that 87% of mothers stated that they would seek help if their child had a mental disorder. However, among them, less than half (43.4%) of mothers of children with a mental disorder, actually consulted. In the analysis of attitudes of mothers towards possible potential structural and cultural barriers to help-seeking, one of our most important findings was that, contrary to the common prejudice that minorities do not seek treatment due to fear of stigma [66, 67], the main obstacles for mothers in our study were those related to access to mental health care, that is, structural factors that may be remedied by the authorities responsible. This finding is in agreement with what was found by Reardon and colleagues [11] in their systematic review, that in the first place, systemic-structural barriers such as accessibility, cost of services, waiting times and getting a referral were the most dominant cultural attitudes related to obstacles to help-seeking. We found that the beliefs that treatment might be detrimental and that there might be reprisal by the authorities if they find out about help-seeking, were ranked in second and third place. In the fourth place mothers mentioned stigma as an obstacle to help-seeking and lastly they mentioned their distrust in professionals.

Our exploratory factor analysis suggests that the factors underlying the CoBaQ are distinct constructs, although in our study population three items in the original scales are cross-loaded to other scales. Of particular interest are our findings that two items which originally were part of the “Accessibility” scale were cross-loaded: ‘lack of information’ was cross-loaded to “Fear of reprisal by authorities” and ‘professionals do not speak Arabic’ was cross-loaded to the “Treatment damages” subscale. This would imply that ‘lack of information’, is perceived by our minority populations as being the fault of the help-seeker rather than that of the service provider. This is an important distinction, with implications for the role of service providers to engage the community and provide information through schools, maternal and child health clinics and HMOs regarding how to access mental health services when needed. The cross-loading of the second item – ‘professionals do not speak Arabic’, which originally belonged to the “Accessibility” scale to the “Treatment damages” scale would indicate that the fact that professionals speak Hebrew and interview the adolescents and their parents in a language that is not their own may be deleterious in terms of miss-diagnosis and/or inappropriate treatment. Language here is perceived as more than a tool for access; it may have powerful potential for damage. Mental health diagnosis and treatment are based on communication between patient and caregiver, and therefore, without the aid of language-free and objective tools, language is of utmost importance.

A third item – ‘public knowledge of treatment may damage future chances’, originally belonging to the “Stigma” scale, was cross-loaded by our minority population to the “Treatment damages” scale. In this case, it is not just a matter of stigma for the subject seeking help but rather concrete future damage that makes mothers prefer to do without.

Our finding concerning the perception that “treatment damages” is in agreement with findings among black ethnic minorities in the USA [68, 69], that report that black and ethnic minority populations are cautious about their contact with mental health care; “… black people are disadvantaged in society, and there is a perception that mental health services reinforce some of these disadvantages, as they believe that mainstream mental health services cannot offer positive help” [68].

When studying the meaning of the different constructs in different cultural contexts and languages, it is also necessary to address questions related to translation. Questionnaires that are translated and used in cultures that are very different from those for which they are originally created, may partly explain the disagreement between our EFA and the original “Perception of Barriers to Service” of the CASA version 5.0 [39]. For one, there might be inherent problems in the scale which measures more heterogeneous content than intended [9]. It is also possible that the translation suffers from lack of semantic equivalence [70] as could be the case if one of the items had a slightly different meaning in Arabic and among these subjects than in English. A third possibility, favored by us, is that different cultural norms, social desirability and experience as a discriminated minority produce different results.

Although of less importance than “Accessibility” for both population groups, a finding that called our attention was that more Druze than Muslim mothers believed “Reprisal by authorities” to be an obstacle to help-seeking. This is a very interesting finding, given that the Druze are much more integrated into Israeli society; their male citizens participate in the military and security forces and they are recognized as partners to the Jewish population. The Muslim population, on the other hand, are perceived as “on-trial citizens”. Thus, despite their somewhat elevated status, Druze mothers believed that an obstacle to help-seeking would be the reprisal by authorities. One possible explanation for this is that Druze youth are enrolled in the Israeli Defense Forces (IDF), and this is the main venue for them to enter the Israeli mainstream and improve their socio-economic status. Since they aim for high ranking positions in the IDF and for future employment in the security forces, they may be wary to jeopardize their future chances by having been diagnosed or labelled with, or treated for a psychiatric problem [71, 72], especially when considering employment-related discrimination [73].

Mothers of adolescents with an externalizing disorder were more likely to perceive obstacles related to accessibility as being very important. When analyzing perceived obstacles to help-seeking according to actual practices of mothers we found that mothers who did consult gave more importance to obstacles related to accessibility and distrust of professionals, and less importance to obstacles related to stigma than mothers who did not consult. It is possible that mothers who did consult actually had a realistic perspective of barriers to help seeking as they experienced difficulties related to accessibility and faced problems related to their trust in the professionals caring for their kin.

### Limitations

One limitation of this study is that the sample includes only school attendants and thus misses drop-outs or non-attenders who usually are a high risk population. However, in Israel, school attendance in the 9th grade is mandatory and therefore in this population drop-out rates are relatively low [74]. Another limitation is that it was not possible for us to register the socio-economic status of each family and thus, have only an estimate of the socio-economic index of the locality in general. For this reason, although we found that “Accessibility” was the main obstacle to help-seeking, we could not assess whether mothers who lived in a locality with a mental health clinic had in fact higher rates of help-seeking than those living in a locality without a mental health clinic, independently of socio-economic and educational characteristics of the family. In our sample religion and socio-economic index were highly correlated (Pearson correlation = .707), as were locality and religion and locality and existence of mental health clinic. It will be of great importance to obtain in the future a better estimate of familial socio-economic level in order to further understand the populations’ choices.

## Conclusions and recommendations

Lack of access to mental health services, rather than cultural obstacles such as stigma, was considered the main obstacle to help-seeking in this Arab minority population. This findings stands in opposition to a common belief among many health professionals and policy makers who believe that even if services were available, the Arabs in Israel would not use them due to stigma. That stigma is not the main obstacle to help seeking is significant because a change in cultural norms requires long-term process involving the population, whereas lack of accessibility to professional services, which is the responsibility of the regulator (the Ministry of Health) and of those providing the care (the HMOs), could be remedied more promptly by a change in policy. It is important to emphasize that this study deals with help-seeking and not with obtaining treatment but we might deduce that the rates of youth in need that do not get treated are even higher. Consequently, a major recommendation is to increase the number of public mental health clinics in the minority localities; to provide appropriate transportation and to make services available in a reasonable period of time. Since lack of knowledge about the existence of treatment was perceived in our study as the fault of the population itself, a further recommendation is the development of information campaigns by the authorities in order to acquaint the minority populations with the different services available. Psychoeducation for parents, who are the gatekeepers and responsible for their children’s mental health service use [38, 75, 76], as well as interventions to address anxiety related to detrimental treatment and stigma may be very helpful in advancing mental health service use among adolescents and their families. As well, psychoeducation for the schools’ educational staff, who could be made aware of the need for referral to more professional mental health services for some students would be beneficial and this would require strengthening the ties between the school staff and the child and adolescent mental health clinics in the vicinity.

In this population, mothers and adolescents seek help mainly from school sources, which have the advantage of being free and available at all times without the need for transportation, and are characterized by a language and cultural fit between students and school staff. There is, therefore, an urgent need to coordinate the government agencies involved in mental health service provision for children and adolescents, so that school mental health services are integrated into the rest of the services and provide quality professional mental health care to adolescents and their families. It is important that policy makers create a plan with clear goals and schedules that must be met in order to reduce the treatment gap, giving preference to population groups where the gap is highest.

## Authors’ information

This work was performed by Raida Daeem as partial fulfillment of the requirements for a PhD degree by the Sackler Faculty of Medicine, Tel Aviv University, Israel.
